# Efficient gene delivery into the embryonic chicken brain using neuron-specific promoters and in ovo electroporation

**DOI:** 10.1186/s12896-022-00756-4

**Published:** 2022-09-02

**Authors:** Kyung Min Jung, Kyung Je Park, Young Min Kim, Jae Yong Han

**Affiliations:** Department of Agricultural Biotechnology, Research Institute of Agriculture and Life Sciences, Seoul National University, 1 Gwanak-ro, Gwanak-gu, Seoul, 08826 Korea

**Keywords:** Chicken, Embryonic brain, In ovo electroporation, Neuron promoter, Gene delivery

## Abstract

**Background:**

The chicken in ovo model is an attractive system to explore underlying mechanisms of neural and brain development, and it is important to develop effective genetic modification techniques that permit analyses of gene functions in vivo. Although electroporation and viral vector-mediated gene delivery techniques have been used to introduce exogenous DNA into chicken embryonic cells, transducing neurons efficiently and specifically remains challenging.

**Methods:**

In the present study, we performed a comparative study of the ubiquitous CMV promoter and three neuron-specific promoters, chicken Ca2+/calmodulin-dependent kinase (cCaMKII), chicken Nestin (cNestin), and human synapsin I. We explored the possibility of manipulating gene expression in chicken embryonic brain cells using in ovo electroporation with the selected promoters.

**Results:**

Transgene expression by two neuron-specific promoters (cCaMKII and cNestin) was preliminarily verified in vitro in cultured brain cells, and in vivo*,* expression levels of an EGFP transgene in brain cells by neuron-specific promoters were comparable to or higher than those of the ubiquitous CMV promoter. Overexpression of the *FOXP2* gene driven by the cNestin promoter in brain cells significantly affected expression levels of target genes, *CNTNAP2* and *ELAVL4*.

**Conclusion:**

We demonstrated that exogenous DNA can be effectively introduced into neuronal cells in living embryos by in ovo electroporation with constructs containing neuron-specific promoters. In ovo electroporation offers an easier and more efficient way to manipulate gene expression during embryonic development, and this technique will be useful for neuron-targeted transgene expression.

**Supplementary Information:**

The online version contains supplementary material available at 10.1186/s12896-022-00756-4.

## Background

The chicken embryo is a preferred animal model for developmental biology, and has also been used to make significant discoveries in cell biology, virology, immunology, cancer biology, epigenetics, and neuroscience [1–5]. The chicken in ovo model can be used as an alternative to primary culture to study the growth, development, and function of neurons that are difficult to propagate. Accordingly, prior studies have conducted neuronal transduction of embryonic chicken brains using recombinant avian adeno-associated viruses (rAAAVs) and the ubiquitous RSV promoter [6]. However, the material access and technical difficulties of the AAAV system [7], safety concerns due to the possibility of genotoxic integration, and unintentional transduction by use of a ubiquitous promoter remain as challenges [6, 8].

The chicken in ovo system can be used in conjunction with electroporation of genetic constructs to perform gene gain- and loss-of-function studies [1, 9–12]. Electroporation enables efficient and relatively localized transfection of regions of interest through strategic selection of injection sites and electrode placement. Further, electroporation circumvents the caveat of receptor interference encountered in viral systems, and nondividing cells can be transduced. Moreover, this technique can be used to deliver plasmids encoding the gene of interest under a cell type-specific promoter [1, 13]. Accordingly, the chicken in ovo system has been extensively used to study retinal development, and electroporation of plasmid constructs has been used for in vivo gain- or loss-of-function studies [9, 14]. By using in ovo electroporation of plasmid DNA into embryonic chicken eyes, Chen et al. demonstrated that c*Nf2* overexpression in lens epithelial cells inhibits cell proliferation [9]. Similarly, Sehgal et al. demonstrated that ectopic expression of *Pax2* in the ventral optic nerve cup prevents choroidal fissure closure [14].

Promoters are a key factor determining the expression dynamics of transgenes, and various promoters are being studied in neuronal cells. There are physicochemical promoters such as electrical, thermal, magnetothermal, NIR, and flash photo catalytic promoters in physical fields, and dopamine, antioxidant, and S-Nitrosylation of histone deacetylase promoters in chemical ones [15–22]. Promoter comparison studies can improve transgene delivery into the brain [23]. Nieuwenhuis et al. performed a comparative study between commonly used promoters (CAG, CMV, PGK, and Syn) in rat and mouse brains using an adeno-associated virus (AAV) system, and identified that the PGK and Syn promoter induces the strongest transgene expression and that Syn is neuron-specific [23]. Radhiyanti et al. also performed a comparative study of both ubiquitous and neuron-specific promoters using an AAV system in the mouse brain, demonstrating that all neuron-specific promoters are active specifically in neurons, and that the NSE promoter induced the highest transgene expression [24]. As such, comparative studies of promoters are essential for effective and specific transgene expression, but the optimal promoter for transgene expression in chicken embryonic brain cells has not yet been determined.

In the present study, we aimed to identify a promoter that is neuron-specific and induces strong transgene expression when delivered by in ovo electroporation. We compared transgene expression under four different promoters, including CMV, chicken CaMKII, chicken Nestin, and human synapsin I. Further, we applied a selected neuron-specific promoter cNestin together with the in ovo electroporation approach to control *FOXP2* target gene expression in vivo. *FOXP2* is the first gene relevant to the human language development [25]. Mutations in *FOXP2* or the expression patterns of *FOXP2* in the developing brain affect severe speech and language disorders [26, 27]. Also, the *FOXP2* gene is important for cortical neurogenesis and cell migration [28] and was therefore selected as a target gene. The present study contributes to the optimization of in ovo electroporation-mediated transgene delivery to chicken embryonic brain cells using neuron-specific promoters, laying the groundwork for future studies of specific gene functions and related mechanisms. This platform can also be used as a model system for brain developmental biology.

## Methods

### Experimental animals

The care and experimental use of chickens were approved by the Institute of Laboratory Animal Resources, Seoul National University, Korea. All procedures, including chicken maintenance, reproduction, and sample collection, were governed by standard operating protocols according to a standard management program at the University Animal Farm, and the Animal Genetic Engineering Laboratory, Seoul National University.

### Culture of primary chicken brain cells and DF-1 fibroblasts

For primary culture of chicken brain cells, embryonic brains of White Leghorn chicks were collected from day 9 eggs, which corresponded to Hamburger and Hamilton (HH) stage 35 [29]. Brains were minced and dissociated with 0.25% trypsin and resuspended in Neurobasal medium (Thermo Fisher Scientific, Waltham, MA, USA) supplemented with 2% B-27 (Thermo Fisher Scientific), 2 mM glutamine (Thermo Fisher Scientific), and 20 ng/ml nerve growth factor (NGF) (MilliporeSigma, Burlington, MA, USA). Culture of primary brain cells was conducted as described previously [30]. Chicken DF-1 fibroblast cells [CRL-12203; American Type Culture Collection (ATCC), Manassas, VA, USA] were maintained and subpassaged in DMEM (Hyclone Laboratories, Logan, UT, USA) supplemented with 10% fetal bovine serum (Hyclone Laboratories) and Antibiotic–Antimycotic solution (Thermo Fisher Scientific). Both primary chicken brain cells and DF-1 fibroblasts were cultured in an incubator at 37 °C with 5% CO_2_ and 60–70% relative humidity.

### RT-PCR analysis

Total RNA samples were isolated with Trizol (Invitrogen, Thermo Fisher Scientific Inc., Carlsbad, CA, USA), and cDNA was synthesized using the Superscript III First-Strand Synthesis System (Invitrogen). RT-PCR was conducted using *PAX6*, *SYP*, and *GAPDH* primers (Additional file 1: Table S1) with conditions as follows: 95 °C 5 min, 35 cycles at 95 °C for 30 s, 60 °C for 30 s, and 72 °C for 1 min.

### Immunocytochemistry

Primary chicken brain cells cultured for 2 weeks and DF1 fibroblast cells were fixed in 4% paraformaldehyde for 10 min, washed three times with PBS, and permeabilized with 0.1% Triton X-100 for 10 min. After washing with PBS, cells were blocked with a blocking buffer (5% goat serum and 1% bovine serum albumin in PBS) for 1 h and then incubated with Alexa Fluor 488 conjugated anti-NeuN antibody (Cat. No. MAB377X, MilliporeSigma) at 4 °C overnight. Following three washes with PBS, cells were mounted with ProLong Gold antifade reagent with DAPI and analyzed under a fluorescence microscope.

### Plasmid construction

The cCaMKII promoter sequence was obtained from genomic DNA of the chicken blastoderm. Genomic DNA was amplified using cCaMKII-specific primers (F: 5′- TGC GCT GCT TCG CGA ACT GCC ACA TCC TTC GAT TTG CCT -3′; R: 5′- ATA GTG AGT CGT ATT CCC AAG GGG CTG GCA ATG C -3′). The PCR product was cloned into the pGEM-T Easy Vector System (Promega, Madison, WI, USA). After sequence verification by Sanger sequencing (Bionics, Seoul, South Korea), recombinant plasmids containing the cCaMKII sequence were reamplified using enzyme site and recipient vector sequence-containing primers (F: 5′- TCG CGA ACT GCC ACA TCC TTC GAT T -3′; R: 5′- AAG CTT GGG TCT CCC TAT AGT GAG TCG TAT TCC CAA GGG GCT GGC AAT GCT -3′). The PCR product was cloned into a pGEM-T Easy Vector and sequence was verified by Sanger sequencing (Bionics). The cNestin and hSynI promoter sequences were synthesized by Bioneer (Daejeon, South Korea). Three promoters were integrated into a *piggyBac* TK Neo^R^ CMV GFP FRT backbone vector generated as described previously [31]. The cCaMKII, cNestin, and hSynI sequences were inserted into the backbone vector using NruI and HindIII restriction enzymes. To construct the *FOXP2* expression vector, the *FOXP2* coding sequence was synthesized by Bioneer and cloned into the *piggyBac* TK Neo^R^ cNestin GFP FRT vector using BsrGI and NotI restriction enzymes.

### Transfection

Primary chicken brain cells cultured for 2 weeks were transfected in serum-free medium with 1–2 μg *piggyBac* EGFP vectors that express EGFP under four different promoters and 1–2 μg of a transposase vector (CAGG PBase), using Lipofectamine 2000 (Invitrogen). The transfection mixture was replaced with culture media 6 h after transfection. Two days after transfection, EGFP expression was observed by fluorescence microscopy. DF1 fibroblasts were also transfected as described above.

### In ovo electroporation

To accurately inject the DNA mixture into the brains or limbs of embryos, recipient chicken embryos were cultured in surrogate eggshells as described previously [32]. Briefly, White Leghorn embryos cultured for 3 days were transferred to recipient eggshells with the embryos facing up, sealed with cling film and egg white, and incubated until embryonic day 5 (E5) without egg turning. In ovo electroporation was performed on the midbrain or right limb. The vitelline membrane was carefully removed using forceps. To introduce plasmid DNA, a glass needle was slowly placed into the brain or limb and used to deliver fast green-positive DNA solution. To electroporate the cells, parallel fixed platinum electrodes were applied to embryos (six pulses, amplitude 25 V, duration 60 ms and interval 100 ms). The eggshell opening was sealed with cling film, and the embryos were returned to the incubator until embryonic day 6 (E6).

### Immunohistochemistry

Embryonic brains or limbs of chicken embryos at embryonic day 6 (E6) were paraffin-embedded and sectioned (thickness, 9–10 μm). After deparaffinization, sections were washed three times with PBS and blocked with blocking buffer (5% goat serum and 1% bovine serum albumin in PBS) for 1 h at room temperature. Sections were then incubated 4 °C overnight with rabbit anti-GFP antibodies diluted 1:200 in blocking buffer. After washing three times with PBS, sections were incubated with Alexa Fluor 488-conjugated secondary antibody (Thermo Fisher Scientific) for 1 h at room temperature. After washing three times with PBS, sections were mounted with ProLong Gold antifade reagent with DAPI and imaged on a confocal fluorescence microscope (Carl Zeiss GmbH, Oberkocken, Germany). Mean EGFP intensity per transfected brain cell was measured using image J software (Wayne Rasband, National Institutes of Health, Bethesda, MD, USA).

### Quantitative RT-PCR

Total RNA samples were isolated from embryonic brains using Trizol (Invitrogen), and cDNA was synthesized using the Superscript III First-Strand Synthesis System (Invitrogen). Gene expression levels were measured using EvaGreen dye (Biotium, Hayward, CA, USA) and a CFX96 Real-Time PCR Detection System (Bio-Rad, Hercules, CA, USA) using the specific primers *FOXP2*, *CNTNAP2*, *ELAVL4*, and *GAPDH* primers (Additional file 1: Table S1). All samples were normalized to internal controls, and fold changes were calculated through relative quantification (2^−△△Ct^).

### Statistical analysis

Statistical analysis was performed using GraphPad Prism 9 (GraphPad Software, CA, USA). Significant differences between groups were determined using Student’s t-tests. Statistical significance was ranked as *p < 0.05; **p < 0.005; ***p < 0.0005; ****p < 0.0001.

## Results

### In vitro* culture and characterization of primary chicken embryonic brain cells*

**Fig. 1:**
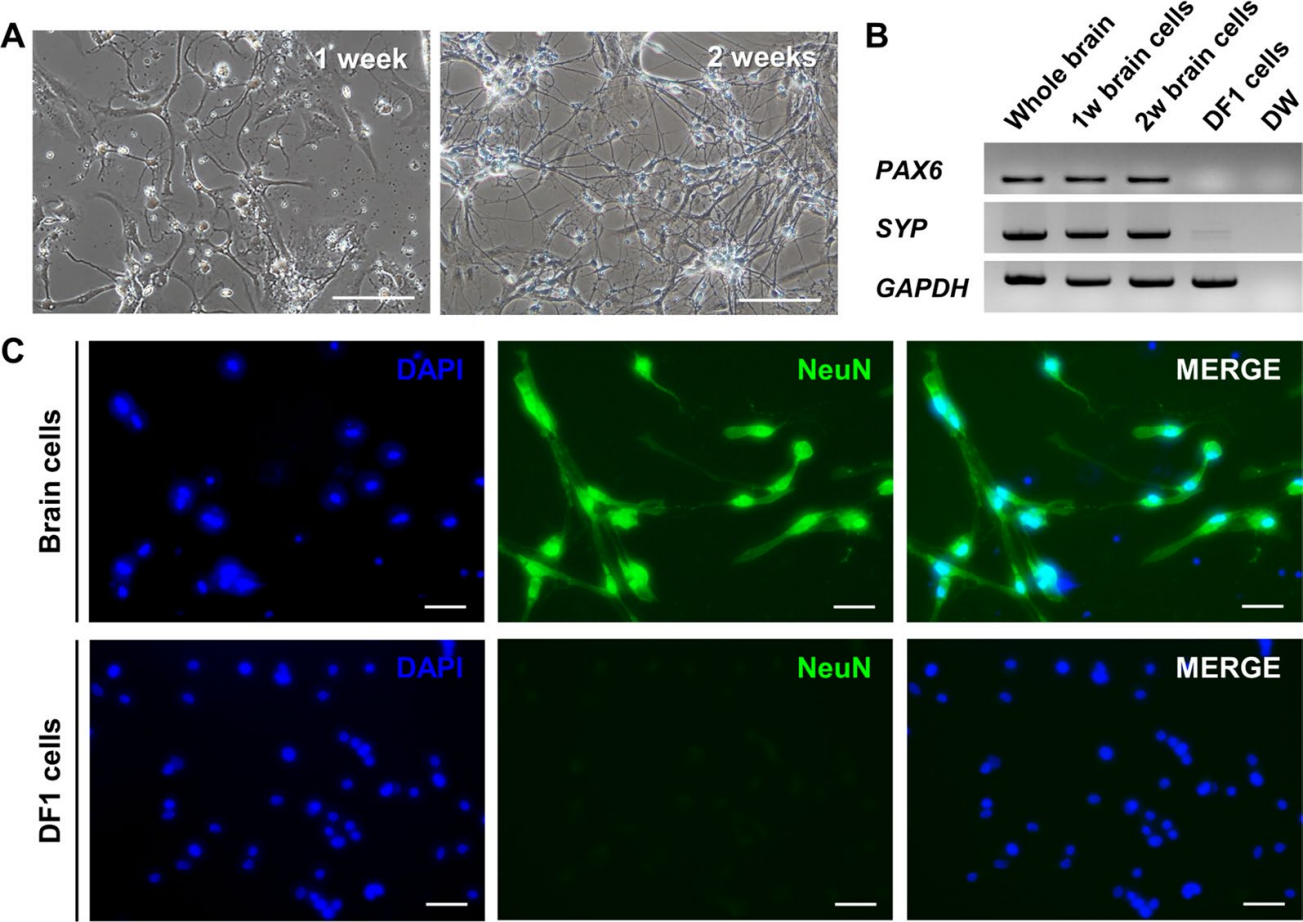
Primary culture and characterization of chicken embryonic brain cells. **A** Morphology of primary cultured chicken brain cells. Scale bar, 200 μm. **B** RT-PCR measurement of *PAX6* and *SYP* expression in primary cultured brain cells. DF1 fibroblast cells were used as a negative control. DW, distilled water. Uncropped gel images are provided in Additional file 2: Fig. S1. **C** Primary brain cells cultured for 2 weeks were immunostained with anti-NeuN antibody. DF1 fibroblast cells were used as a negative control. Scale bar, 50 μm

Whole brain cells were isolated from chicken embryos at Hamburger–Hamilton (HH) stage 35 and cultured for 2 weeks in vitro as described previously [30] (Fig. 1A), and cells were then characterized. RT-PCR analysis revealed that primary cultured chicken embryonic brain cells expressed the neuron-specific genes *PAX6* and *SYP*, and that expression was maintained for more than 2 weeks (Fig. 1B and Additional file 2: Fig. S1). Further, immunostaining with an anti-NeuN antibody revealed that the cells were positive for a universal neuronal marker (Fig. 1C). DF1 fibroblast cells used as negative controls did not express neuron-specific genes or NeuN protein. These results demonstrated that chicken embryonic brain cells could be cultured in vitro and used for subsequent neuron-specific promoter testing in vitro.

### *Investigation of neuron-specific promoters in chicken embryonic brain cells *in vitro

**Fig. 2:**
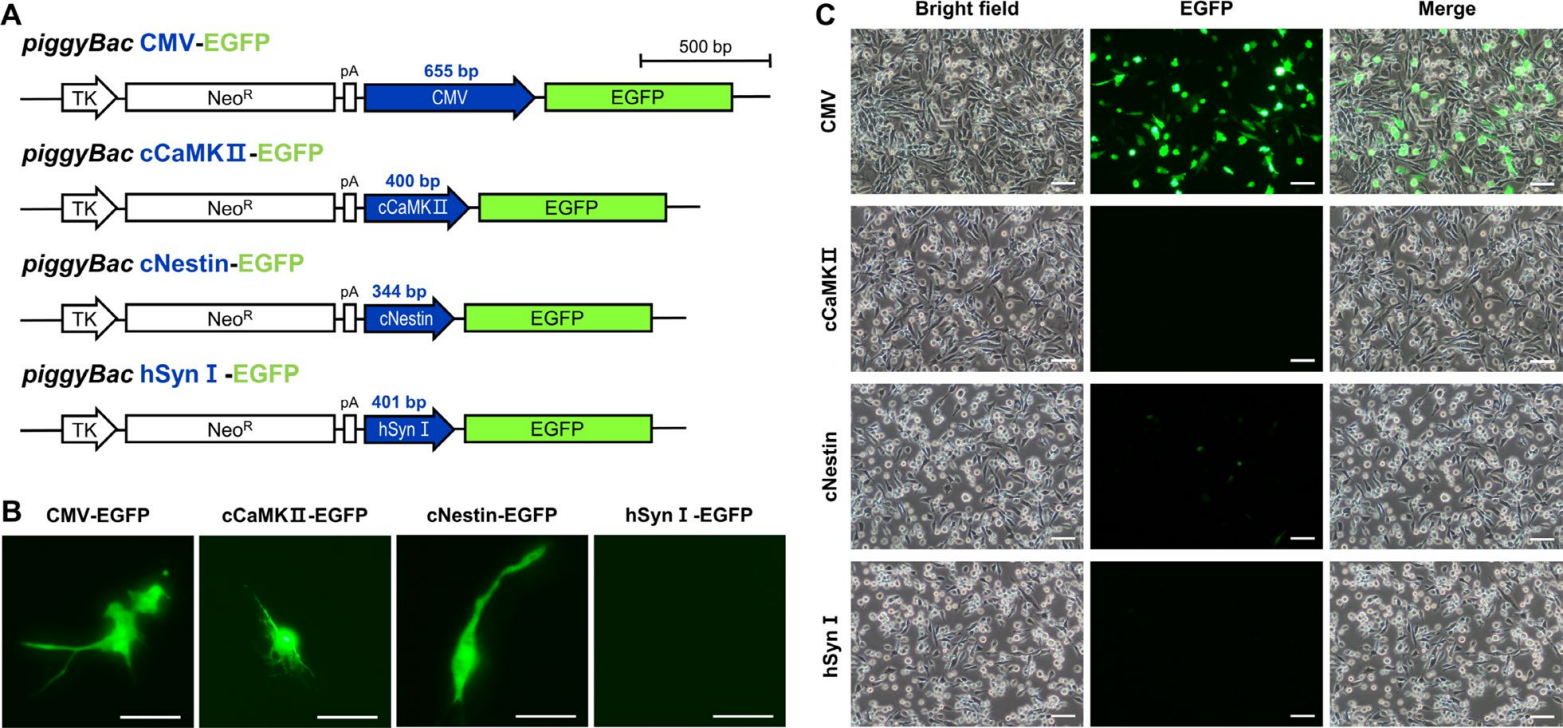
Neuron-specific promoters in chicken primary brain cells in vitro. **A** Schematic representation of *piggyBac* vectors, which express enhanced green fluorescent protein (EGFP, 720 bp) under four different promoters. The CMV promoter is 655 bp. The chicken CaMKII (cCaMKII) promoter is 400 bp. The chicken Nestin (cNestin) promoter is 344 bp. The human Synapsin I (hSynI) promoter is 401 bp. bp, base pairs. **B** Transfected primary chicken brain cells. Two days after transfection, EGFP expression was visualized by fluorescence microscopy. Scale bar, 50 μm. **C** DF1 cell line transfected with four vectors expressing EGFP under different promoters. Scale bar, 100 μm

To investigate the neuron-specific promoters available in chickens, we constructed four vectors to express EGFP under different promoters (one ubiquitous promoter, CMV; three neuron-specific promoters, chicken CaMKII, chicken Nestin, and human synapsin I) and transfected them into cultured chicken embryonic brain cells (Fig. 2A). Transient EGFP signals due to delivery of the EGFP-coding sequence by the *piggyBac* vector were detected in chicken embryonic brain cells 2 days after transfection. EGFP-expressing cells were observed in cells transfected with vectors containing CMV, cCaMKII, or cNestin promoters, while EGFP was not detected in cells transfected with the vector containing the hSynI promoter (Fig. 2B). Additionally, we applied four vectors to DF1 fibroblast cells to investigate the promoters’ cell type specificity. EGFP expression via the CMV promoter was strongly detected with high efficiency, while EGFP expression via neuron-specific promoters was not detected in DF1 fibroblast cells, with exception to a few cells expressing EGFP via the cNestin promoter (Fig. 2C). Collectively, these findings demonstrated that CMV, cCaMKII, and Nestin promoters could be used to introduce foreign genes into chicken embryonic brain cells, and that the cCaMKII and cNestin promoters were specific to brain cells.

### *Comparative analysis of targeted EGFP expression between promoters in chicken embryonic brain cells using *in ovo* electroporation*

**Fig. 3:**
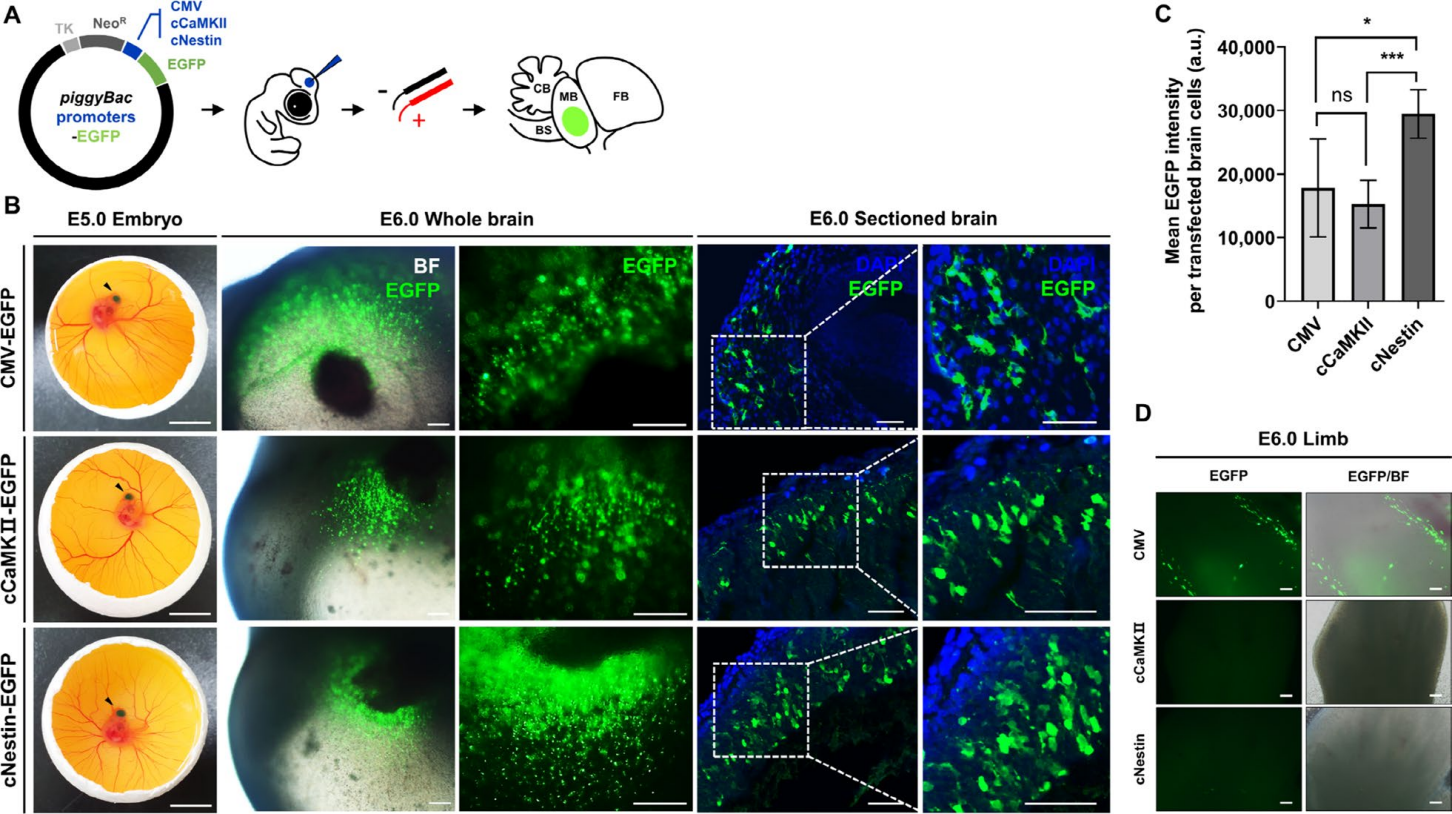
In vivo test of selected neuron-specific promoters. **A** Schematics of the vector constructs and injections. The blue dot indicates the injection region. FB, forebrain; MB, midbrain; BS, brainstem; CB, cerebellum. **B** Promoter test with in ovo electroporation and surrogate culture. EGFP expression induced by four different promoters was analyzed in the embryonic brain on embryonic day 5 (E5). One day after electroporation, EGFP expression was observed by fluorescence microscopy in the injected region of the whole brain. The brain was paraffin-sectioned and stained with anti-EGFP antibody. The CMV promoter was used as positive control. The dotted box indicates the region of magnification image. Scale bar, 1 cm (embryos), 200 μm (whole brain), and 50 μm (sectioned brain). **C** Quantification of mean EGFP intensity per transfected brain cell by ImageJ. Significant differences between groups were determined using Student’s t-tests. ns, not significant; *p < 0.05; **p < 0.005; ***p < 0.0005. Embryos n = 5. **D** Promoter specificity test by in ovo electroporation of embryonic limbs. One day after electroporation, EGFP expression was observed by fluorescence microscopy in the injected region of limbs. Scale bar, 200 μm. Embryos n = 3

To determine the feasibility of inducing targeted expression of EGFP in the brain cells of a developing chicken embryo, we introduced the exogenous EGFP gene using in ovo electroporation with the promoters tested in vivo (CMV, cCaMKII, and cNestin). EGFP-expressing plasmid DNA was injected into the midbrains of embryos, and electric pulses were applied. After 24 h of incubation, EGFP signal was expressed in only injected region of the brain and was not observed in other embryonic tissues (Fig. 3A, B). To compare the intensity of EGFP expression induced by each promoter, average EGFP intensity per transfected brain cell in sectioned brain images was measured using image J software. The CMV and cCaMKII promoters induced similar EGFP intensity, and the cNestin promoter induced EGFP with a significantly higher intensity than other promoters (Fig. 3C). Additionally, we applied three vectors, each containing a different promoter, to embryonic limbs to investigate promoter tissue specificity. At 24 h after in ovo electroporation of limbs, EGFP-expressing cells were present in the limbs of the CMV promoter group, while EGFP-expressing cells were not detected in the cCaMKII promoter group, and a few EGFP-expressing cells were observed in the cNestin promoter group (Fig. 3D). These findings suggest that foreign genes can be effectively transferred into the embryonic brains of developing chickens using the in ovo electroporation method, and that the cCaMKII and cNestin promoters can act specifically on brain cells.

### *Use of *in ovo* electroporation and neuron-specific promoters to study *in vivo* gene function*

**Fig. 4:**
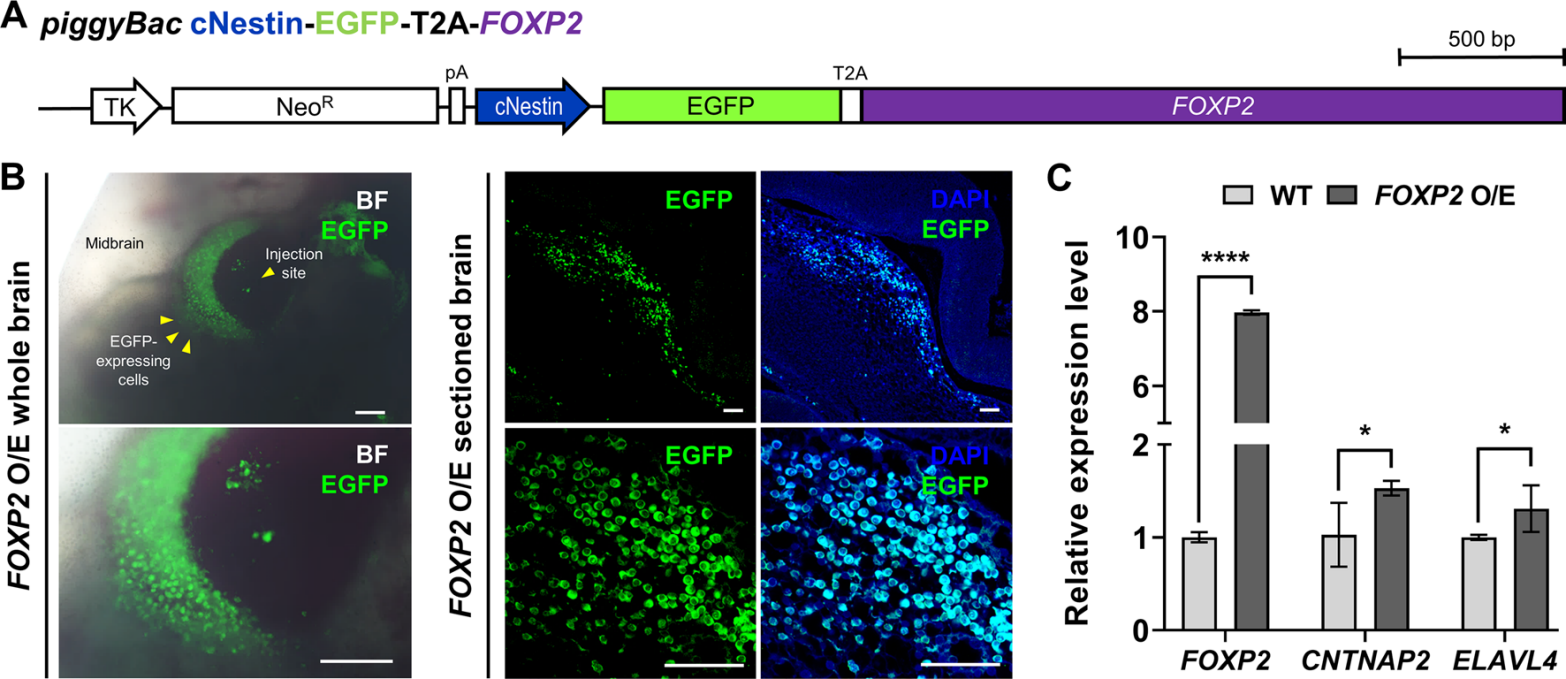
Ectopic expression of *FOXP2* in chicken embryonic brain cells increases *ELAVL4* and *CNTNAP2* expression. **A** Structure of *FOXP2*-overexpressing plasmid. **B** *FOXP2*-overexpressing vector test using the in ovo electroporation and surrogate culture system. EGFP expression was analyzed by cNestin promoter in the embryonic brain at embryonic day 5 (E5). One day after electroporation, EGFP expression was observed by fluorescence microscopy in the injected region of the whole brain. Scale bar, 200 μm. EGFP expression was also observed by immunohistochemistry in paraffin sections using anti-GFP antibody. Scale bar, 200 μm (whole brain) and 50 μm (sectioned brain). **C** Quantitative RT-PCR analysis of *FOXP2*, *CNTNAP2*, and *ELAVL4* genes in wild-type (WT) and *FOXP2*-overexpressing brain. Significant differences between groups were determined using Student’s t-tests. *p < 0.05, ****p < 0.0001. Embryos n = 4

Subsequently, we utilized neuron-specific promoters and the in ovo electroporation method, to control expression of specific genes and determine their effects in the chicken embryonic brain in vivo. We introduced plasmid DNA expressing *FOXP2* simultaneously with EGFP under the cNestin promoter into the embryonic brain through in ovo electroporation (Fig. 4A). EGFP expression was detected in the injected region of the brain (Fig. 4B). Quantitative RT-PCR analysis of *CNTNAP2* and *ELAVL4*, showed that both genes were upregulated with overexpression of *FOXP2* (Fig. 4C). In conclusion, these findings suggest that this platform could be applied to gene function studies related to neurobiology. Using this method, neuron-specific target gene expression can be induced in the embryonic chicken brain using the in ovo electroporation method in combination with neuron-specific promoters.

## Discussion

The chicken embryonic brain is a valuable model system for investigating the molecular mechanisms underlying neural and brain biology, and it is important to develop effective gene transfer techniques that allow study of gene function and regulation in vivo. Accordingly, in ovo electroporation is well-established and has long been used as an efficient approach to introduce DNA into chicken embryonic tissues [1, 9, 10, 12]. In the present study, we demonstrated the feasibility of this method for expression of exogenous DNA in chicken embryonic brain cells, and examined the transfection specificity and efficiency of the neuron-specific cCaMKII, cNestin, and hSynI promoters.

As a result of introducing EGFP into the chicken embryonic brain via in ovo electroporation-mediated gene transfer, expression of the EGFP transgene driven by the neuron-specific promoters cCaMKII and cNestin was similar to or higher than that of the ubiquitous CMV promoter in vivo. Importantly, the cNestin promoter had greater efficiency than other promoters. Similarly, in a prior study in mice, GFP intensity induced by four different promoters (CBh, mCaMKII, NSE, and SynI) was compared in the whole brain, and the NSE promoter induced GFP expression most efficiently [24]. However, the cNestin promoter induced modest nonspecific expression in nontarget cells (Figs. 2C and 3D). The neuroepithelial stem cell protein Nestin is a cytoskeletal intermediate filament, and is widely used as a marker for neural stem/progenitor cells (NSCs/NPCs) in the developing and adult central and peripheral nervous systems [33–35]. Although the Nestin promoter is highly active in NSCs in the adult brain, off-target transgene expression, such as in ependymal cells, has also been reported [36]. Also, *nestin* expression is not restricted to NSCs, as it has also been detected in myogenic precursor cells, tooth buds, limb buds, heart, testis, and eyes [35]. Therefore, although the expression level of the transgene under the cCaMKII promoter was lower than that of cNestin, cCaMKII could be preferable when specificity is necessary.

We used the commonly used CMV and hSynI promoter sequences based on previous studies [37, 38]. The CaMKII and Nestin promoters had not been previously reported in chickens, and were designed based on chicken genome sequences and previous studies in other species (Additional file 3: Fig. S2) [35, 39]. The promoters used in the present study successfully expressed the EGFP transgene, but further promoter analysis studies are needed to investigate the regulatory elements and specific promoter region for maximum efficiency. For example, by injecting lentiviral vectors into the mouse cerebral cortex, three CaMKII promoter regions (0.4, 1.3, and 2.4 kb) were compared, all of which restricted expression to cortical pyramidal neurons, with the 1.3 kb promoter being the strongest [39]. Cheng et al. performed promoter analysis of the mouse *nestin* gene by testing eight different fragments, determining that the promoter activity of the mouse *nestin* gene resides in the 161 to + 183 bp region [35]. As such, by investigating regulatory elements through promoter analysis of neuron-specific promoters in chicken cells, promoter sequences for in vivo transgene delivery can be further optimized. Also, in addition to the promoters identified in the present study, other potential promoters such as PGK and NSE should be further evaluated in future studies. Additionally, we showed that the expression of both *CNTNAP2* and *ELAVL4*, which are representative target genes of *FOXP2* transcription factor [40, 41], was upregulated by overexpression of *FOXP2* using cNestin promoter and in ovo electroporation. This suggests that this platform can be applied to the study of gene function related to neurobiology.

The present study optimized in ovo electroporation-mediated transgene delivery to neuronal cells in the chicken embryonic brain, providing a basis for future studies of gene function and regulation. In addition, this study laid the foundation for inducing specific expression of transgenes or gene editing in brain cells in the future by identifying a neuron-specific promoter that can replace or surpass the ubiquitous promoter in the chicken embryonic brain. The in ovo electroporation system is an efficient and effective approach, and through combination with specific promoters, this platform can be used for various research including selective manipulation of gene expression in vivo.

## Supplementary Information

**Additional file 1: Table S1**. List of primers used for research.

**Additional file 2: Fig S1**. RT-PCR analysis of cultured embryonic brain cells. Expression of *PAX6* and *SYP* were analyzed in primary cultured brain cells. Whole brain was used as a positive control and DF1 fibroblast cells were used as a negative control. DW, distilled water. The parts shown in Fig. 1B are indicated by black dashed lines.

**Additional** **file** **3**:** Fig** **S2**. Gene structure and promoter sequences of chicken CaMII and Nestin. Dark blue bars indicate promoter regions and light blue bars indicate exon regions. Black bars indicate adjacent genes. TSS, transcription start site.

## Data Availability

The datasets generated and/or analyzed during the current study available from the corresponding author on reasonable request.
