# Supplementary material for: Efficient gene delivery into the embryonic chicken brain using neuron-specific promoters and in ovo electroporation
Source: BMC Biotechnol. 2022 Sep 2;22:25. doi: 10.1186/s12896-022-00756-4 (PMC9440574; doi:10.1186/s12896-022-00756-4)
Supplement: Supplementary file 1 — Additional file 1: Table S1. List of primers used for research. [file 12896_2022_756_MOESM1_ESM.docx]

**Supplementary table 1. List of primers used for research**

| **Gene** | **Primer sequence (5’→3’)** | | **Size (bp)** | **Usages** |
| --- | --- | --- | --- | --- |
| *PAX6* | F: | TTC ACC ATG GCC AAC AAC CT | 235 | RT-PCR |
|  | R: | CAG GTT CAC TGC CAG GAA CT |  |  |
| *SYP* | F: | CCT ACC CGT TCA GGT TGC AT | 533 |  |
|  | R: | GTT GTA GCC ACT GGA CTG CT |  |  |
| *GAPDH* | F: | CAC AGC CAC ACA GAA GAC GG | 443 |  |
|  | R: | CCA TCA AGT CCA CAA CAC GG |  |  |
| *FOXP2* | F: | CCT GGC TGT GAA AGC GTT TG | 104 | Quantitative  RT-PCR |
|  | R: | ATT TGC ACC CGA CAC TGA GC |  |  |
| *CNTNAP2* | F: | GAA GGC AAG GTC AGC GTT CA | 108 |  |
|  | R: | GAA TCG AAC TTC ATG CCA CTG |  |  |
| *ELAVL4* | F: | TCA AGG TGA TCC GCG ACT TC | 103 |  |
|  | R: | CCA TTA AGG CTG GCA ATC GC |  |  |
| *GAPDH* | F: | ACT GTC AAG GCT GAG AAC GG | 99 |  |
|  | R: | ACC TGC ATC TGC CCA TTT GA |  |  |
