# Supplementary material for: Efficient gene delivery into the embryonic chicken brain using neuron-specific promoters and in ovo electroporation
Source: BMC Biotechnol. 2022 Sep 2;22:25. doi: 10.1186/s12896-022-00756-4 (PMC9440574; doi:10.1186/s12896-022-00756-4)
Supplement: Supplementary file 2 — Additional file 2: Fig S1. RT-PCR analysis of cultured embryonic brain cells. Expression of PAX6 and SYP were analyzed in primary cultured brain cells. Whole brain was used as a positive control and DF1 fibroblast cells were used as a negative control. DW, distilled water. The parts shown in Fig. 1B are indicated by black dashed lines. [file 12896_2022_756_MOESM2_ESM.docx]

**
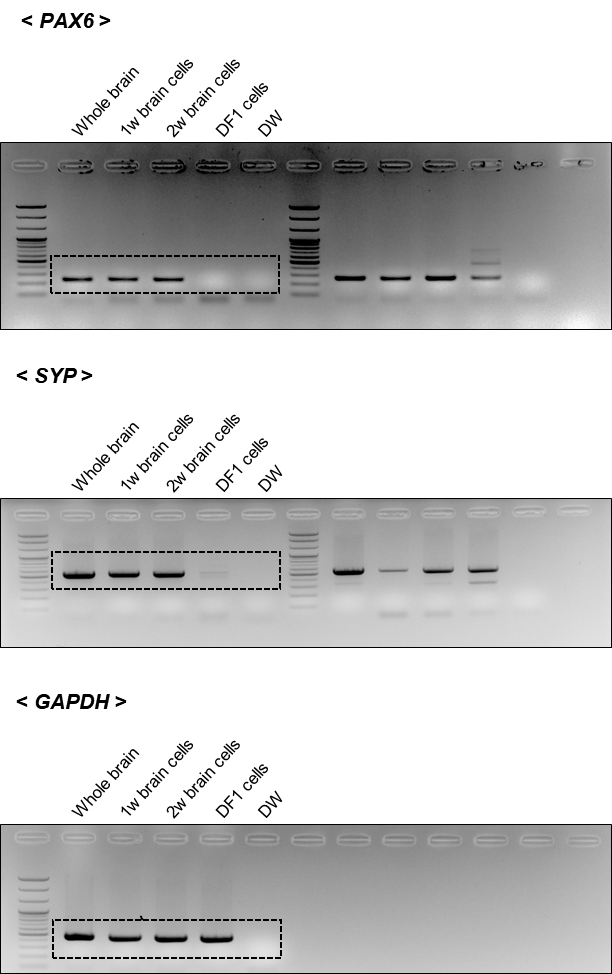
**

**Supplementary figure 1. RT-PCR analysis of cultured embryonic brain cells.** Expression of *PAX6* and *SYP* were analyzed in primary cultured brain cells. Whole brain was used as a positive control and DF1 fibroblast cells were used as a negative control. DW, distilled water. The parts shown in Fig. 1B are indicated by black dashed lines.
