# Supplementary material for: Efficient gene delivery into the embryonic chicken brain using neuron-specific promoters and in ovo electroporation
Source: BMC Biotechnol. 2022 Sep 2;22:25. doi: 10.1186/s12896-022-00756-4 (PMC9440574; doi:10.1186/s12896-022-00756-4)
Supplement: Supplementary file 3 — Additional file 3: Fig S2. Gene structure and promoter sequences of chicken CaMII and Nestin. Dark blue bars indicate promoter regions and light blue bars indicate exon regions. Black bars indicate adjacent genes. TSS, transcription start site. [file 12896_2022_756_MOESM3_ESM.docx]

**
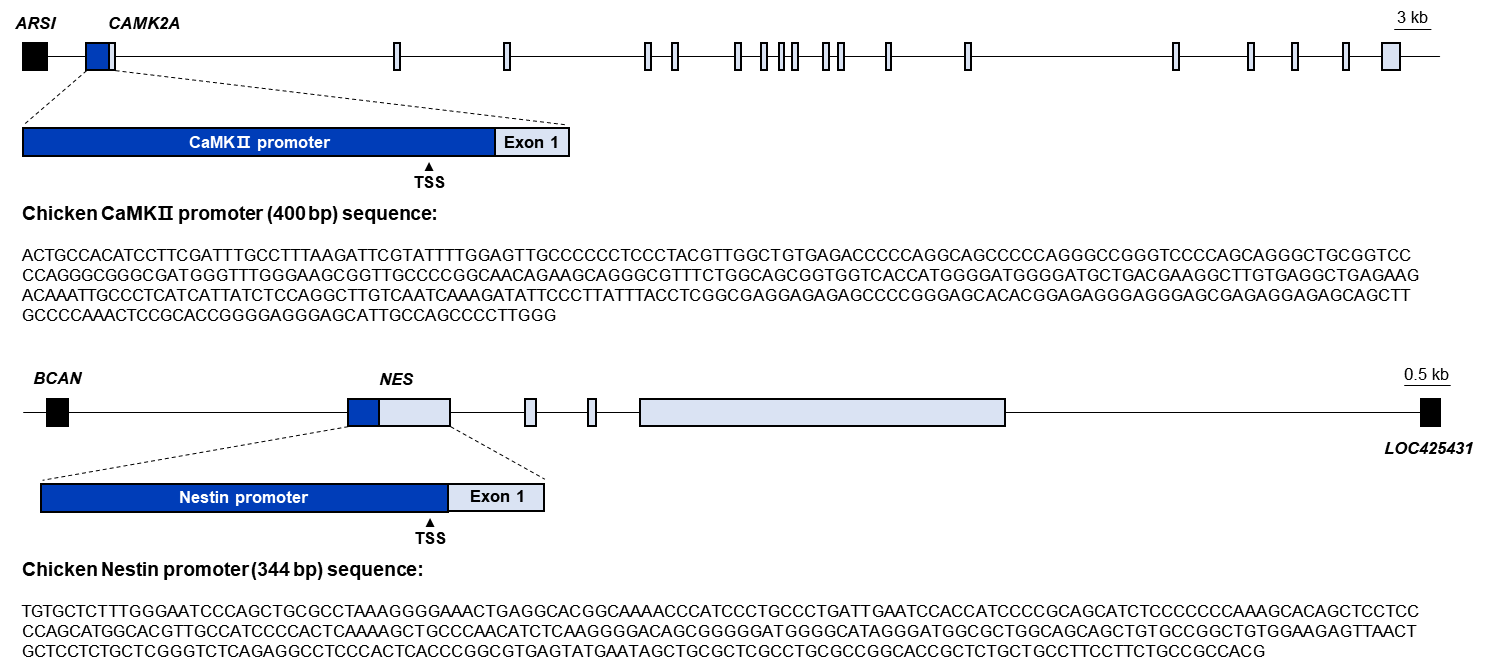
Supplementary figure 2. Gene structure and promoter sequences of chicken CaMKⅡ and Nestin.** Dark blue bars indicate promoter regions and light blue bars indicate exon regions. Black bars indicate adjacent genes. TSS, transcription start site.
